# New advances in prediction and surveillance of preeclampsia: role of machine learning approaches and remote monitoring

**DOI:** 10.1007/s00404-022-06864-y

**Published:** 2022-12-25

**Authors:** Max Hackelöer, Leon Schmidt, Stefan Verlohren

**Affiliations:** Department of Obstetrics, Charité – Universitätsmedizin Berlin, corporate member of Freie Universität Berlin and Humboldt- Universität Zu Berlin, Charitéplatz 1, 10117 Berlin, Germany

**Keywords:** Artificial intelligence, Machine learning, Remote monitoring, Preeclampsia, Hypertensive pregnancy disorders, Angiogenic factors, Multivariable modeling, Decision trees

## Abstract

Preeclampsia, a multisystem disorder in pregnancy, is still one of the main causes of maternal morbidity and mortality. Due to a lack of a causative therapy, an accurate prediction of women at risk for the disease and its associated adverse outcomes is of utmost importance to tailor care. In the past two decades, there have been successful improvements in screening as well as in the prediction of the disease in high-risk women. This is due to, among other things, the introduction of biomarkers such as the sFlt-1/PlGF ratio. Recently, the traditional definition of preeclampsia has been expanded based on new insights into the pathophysiology and conclusive evidence on the ability of angiogenic biomarkers to improve detection of preeclampsia-associated maternal and fetal adverse events.

However, with the widespread availability of digital solutions, such as decision support algorithms and remote monitoring devices, a chance for a further improvement of care arises. Two lines of research and application are promising: First, on the patient side, home monitoring has the potential to transform the traditional care pathway. The importance of the ability to input and access data remotely is a key learning from the COVID-19 pandemic. Second, on the physician side, machine-learning-based decision support algorithms have been shown to improve precision in clinical decision-making. The integration of signals from patient-side remote monitoring devices into predictive algorithms that power physician-side decision support tools offers a chance to further improve care.

The purpose of this review is to summarize the recent advances in prediction, diagnosis and monitoring of preeclampsia and its associated adverse outcomes. We will review the potential impact of the ability to access to clinical data via remote monitoring. In the combination of advanced, machine learning-based risk calculation and remote monitoring lies an unused potential that allows for a truly patient-centered care.

## What does this study add to the clinical work

**Table:** 

Machine learning-based algorithms for early detection of adverse events in pregnant women with preeclampsia are at least equivalent and in some cases superior to conventional methods, e.g., those based on biomarkers such as sFlt-1 and PlGF. Furthermore, the integration of remote monitoring of pregnant women at increased risk into the clinical routine has the potential to additionally improve care, treatment and safety of patients at home.	

## Introduction—what is the scope of this review?

Preeclampsia, a multisystem disorder in pregnancy, is a major cause of potentially preventable maternal and fetal morbidity and mortality [1]. Preeclampsia affects 3–5% of all pregnant women with an upward trend. Worldwide, the disease causes 500,000 fetal and newborn deaths yearly as well as approximately 46,000 maternal deaths, with the majority of these in the low- and middle-income countries [2]. Preeclampsia and its associated outcomes are among the leading causes of maternal morbidity and mortality in developed countries. In high resource settings, many obstetric complications, such as maternal deaths due to unsafe abortions, obstructed labor, peri-partum hemorrhage or sepsis, can be managed and are less likely to happen as compared to regions with less resources [3]. Preeclampsia has been shown to contribute disproportionately to maternal deaths in developed countries. In the Eighths Report of the Confidential Enquiries into Maternal Deaths in the United Kingdom, Preeclampsia, contributing 22 deaths, was the second most frequent cause of direct maternal deaths. Sepsis, which included deaths in early pregnancy due to sepsis, was the most common cause [4]. Of those 22 deaths, 20 involved substandard care and 63% of these were described as “undoubtedly avoidable”. In the report analyzing maternal deaths in the United Kingdom in the triennium 2006 to 2008, the most common cause of maternal death was intracerebral hemorrhage (9 of 22 cases), which is likely to be preventable by antihypertensive medication. In a proportion of these cases, severe hypertension was neither identified nor treated [1]. In the USA, maternal mortality is on the rise, while it declines elsewhere [5]. The maternal mortality ratio, defined as maternal deaths per 100,000 live births, increased from 16.9 in 1990 to 26.4 in 2015 while it decreased in the same timeframe in other high-income countries, such as Australia (7.5–5.5), France (16.9–7.8) or Germany (20.2–9). In approximately the same timeframe, rates of severe preeclampsia have risen in the USA. In comparison to 1980, women delivering in 2003 were at 6.7-fold increased risk of severe preeclampsia [6]. And although maternal mortality is fortunately decreasing in the above-mentioned countries, new challenges are nevertheless emerging for Germany, the U.S. and the rest of the world. Especially since preeclampsia, with its various manifestations, represents a significant personal and financial burden on healthcare systems [7]. Unnecessary hospitalizations of pregnant women with mild to moderate preeclampsia propose a real challenge. Therefore, not only new preventive measures need to be further explored, but also those that help to better assess the individual risk and necessity for immediate treatment thus preventing unnecessary hospitalisations. Analyzing big data with means of machine learning-based approaches holds untapped opportunities, along with remote monitoring methods, to improve care and treatment [8].

Particularly in case of preeclampsia, since there is still no causative therapy other than the delivery of the child, interventions, such as induction of fetal lung maturation, transfer to a perinatal care center, and timed birth, have been shown to contribute to lowering maternal and fetal adverse events [2]. For this reason, a precise prediction of women at risk as well as subsequent tailored antenatal monitoring of mother and child is of considerable importance.

Next to its impact on maternal and fetal/neonatal morbidity and mortality, improving prediction of preeclampsia has a huge health–economic dimension. For the USA, it has been shown that preeclampsia causes an additional annual health-care spending of 2.18 billion US dollar [9]. The presence of preeclampsia (according to the traditional definition) increased the probability of an obstetric adverse event from 4.6 to 10.1% for mothers and from 7.8 to 15.4% for infants while lowering gestational age by 1.7 weeks. Furthermore, when calculating the associated costs based on a combination of population-based and administrative data sets, Stevens et al. showed that the total cost burden of preeclampsia during the first 12 months after birth was $1.03 billion for mothers and $1.15 billion for infants. As expected, and in accordance with clinical observations, the cost burden per infant correlates with gestational age, ranging from $150,000 at 26 weeks gestational age to $1311 at 36 weeks gestational age. This shows the high unmet need to improve prediction of the disease to be able to prevent maternal and fetal morbidity and avoid associated costs.

The scope of this article is to review current advances in prediction and monitoring in case of suspected or manifest preeclampsia in pregnancy. There have been considerable advances in first-trimester screening of the disease. The success in early screening strategies lies in the application of multivariable modeling techniques. We will touch upon these screening approaches briefly. However, we will focus on the yet unmet medical need to increase accuracy of short-term prediction of the disease and especially its adverse outcomes later in pregnancy. When women present with clinical suspicion at high risk, current preeclampsia diagnostic work-up still relies heavily on the clinicians’ observations and an ensemble of diagnostic tools, such as laboratory parameters, biomarkers, and ultrasound findings. These clinical work-up pathways yet make little use of advanced statistical methods generally referred to as machine-learning (ML). We will summarize the advances in short-term prediction algorithms based on machine-learning approaches. Furthermore, access to clinical information via remote monitoring is a promising new field of research which we will review here. In the combination of advanced risk calculation and remote monitoring lies an unused potential as we will delineate in this review.

## What are we trying to predict?

The aim of the clinical definition of preeclampsia is first to identify women at risk of adverse outcomes and second to determine appropriate management to prevent them. For many years, preeclampsia has been defined as the new onset of hypertension and proteinuria after 20 weeks of gestation [10]. These two clinical features have been used as a “screening test” for preeclampsia-associated adverse outcomes. However, it has been shown more than 20 years ago that this “gold standard”, traditional definition of preeclampsia has a low positive predictive value to predict preeclampsia-related adverse outcomes of just 20–30% [11]. This has led to a re-thinking of how to best describe the disease.

### Adverse outcomes

Preeclampsia is a hypertensive pregnancy disorder (HPD). Hypertension in pregnancy is defined as blood pressure values ≥ 140/ ≥ 90 mmHg. Gestational hypertension is the most common hypertensive pregnancy disorder and is defined by the new onset of hypertension (systolic blood pressure ≥ 140 mmHg and/or diastolic blood pressure ≥ 90 mmHg) at ≥ 20 weeks of gestation in the absence of new signs of end-organ dysfunction or proteinuria [12]. Gestational hypertension occurs in roughly 6–17% [13–15] of healthy nulliparous pregnant women and is highest in pregnant women with preeclampsia in a previous pregnancy, multifetal gestation or obesity [15, 16]. Pregnant women with gestational hypertension are at an increased risk of developing preeclampsia. It can be assumed that approximately 10–50% of pregnant women with gestational hypertension develop preeclampsia in the further course of pregnancy leading to a global incidence rate of 2–5% [6, 17, 18].

Preeclampsia-associated adverse outcomes comprise a wide spectrum of maternal and fetal complications. They range from the worst-case event, the maternal and/or fetal/neonatal death to specific end-organ damage, such as pulmonary edema, acute kidney failure, HELLP-syndrome, eclampsia and more on the maternal side. On the fetal side, prematurity and its complications, such as necrotizing enterocolitis (NEC) or intraventricular hemorrhage (IVH), and small for gestational age (SGA) comprise the most frequent complications. While most cases are late-onset cases after 34 weeks of gestation, women with early-onset disease have a higher frequency of maternal and fetal adverse outcomes [19].

In the past decade, an increasing number of studies [20–22] focused on the endpoint “preeclampsia-associated adverse outcomes” with the goal to surpass the proxy “hypertension and proteinuria” and directly identify the outcome of interest. Evaluation of biomarkers, algorithms and even potential treatments for preeclampsia have reported many different results with respect to the accuracy of the intervention tested which is partially due to variations in the exact components feeding the mostly composite endpoint “adverse outcomes”. Therefore, there is a need to harmonize the outcome “adverse outcome” to allow for comparability of studies.

Recently, an international group of healthcare professionals, researchers, and women with lived experience of preeclampsia have developed a core-outcome set of preeclampsia. In a structured approach, including a systematic review of studies, a Delphi consensus followed by stakeholder consultation identified a total of 14 maternal and 8 fetal adverse outcomes (Table 1) [23].

**Table 1:** Core maternal and fetal outcome sets representing severe morbidity or mortality in the context of preeclampsia according to Duffy et al. [23]

Maternal outcomes	Fetal outcomes
Maternal mortality	Stillbirth
Eclampsia	Gestational age at delivery
Stroke	Birthweight
Pulmonary edema	Small-for-gestational-age
Acute kidney injury	Neonatal mortality
Raised liver enzymes	Neonatal seizures
Liver capsule hematoma or rupture	Admission to neonatal unit required
Placental Abruption	Respiratory support
Postpartum hemorrhage	
Low platelets	
Cortical blindness	
Retinal detachment	
Admission to intensive care required	
Intubation and mechanical ventilation	

### The pathophysiology of the disease and its impact on the definition

**Fig. 1:**
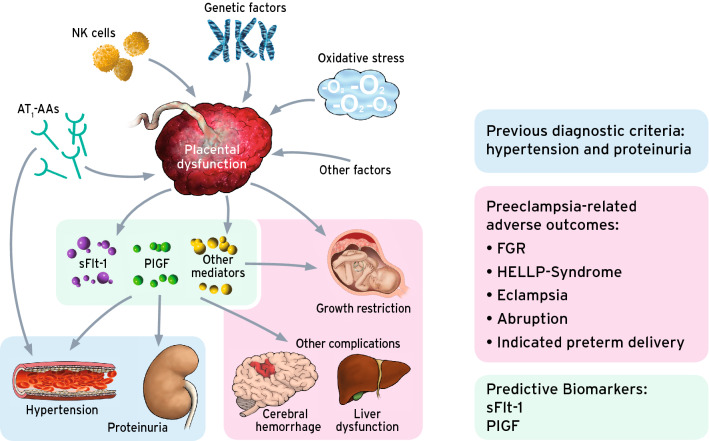
The current understanding of the pathophysiology of preeclampsia: The etiology of the disease and its adverse outcomes centers around a dysfunctional placenta. It is unclear to which extent genetic, immunologic, environmental and other factors and processes contribute to the initial lesion which is located in the placenta. However, it is now clear, that the maternal and fetal symptoms are downstream to an altered expression of angiogenic and antiangiogenic factors. Three findings are key to the clinical results of this concept: 1. The previous diagnostic criteria are at the end of a long pathophysiologic cascade and are only two of many end-organ damages 2. The maternal and fetal adverse outcomes are other effects and the outcome of interest as they are the drivers of maternal and fetal morbidity and mortality. A direct prediction of the complications is desirable. 3. The angiogenic and antiangiogenic factors are deeply rooted in the pathophysiological cascade and aid in better predicting and diagnosing the outcomes of placenta-dysfunction-related events. *NK* natural killer cells, AT1-AAs Angiotensin II Type 1 Receptor Agonistic Autoantibody’s, *sFlt-1* soluble fms-like tyrosine kinase-1, *PlGF* Placental growth factor, *FGR* Fetal growth restriction, *HELLP-*Syndrome Hemolysis, elevated liver enzymes, low platelets

The pathophysiology of the disease is still poorly understood, and a detailed discussion is beyond the scope of this review. Nonetheless, there are three aspects that must be highlighted to understand the current approach to preeclampsia prediction and diagnosis:

1. Hypertension and proteinuria are only two of many clinical features of preeclampsia. They arise at the end of a long pathophysiological cascade, as per definition preeclampsia only occurs after 20 weeks of gestation. The “initial lesion”, involving a disturbed placentation, occurs in the first and early second trimester. The consequence, the maternal syndrome, affecting the whole body of the mother, occurs later (24).
2. The long-standing definition of preeclampsia “hypertension and proteinuria” has a poor correlation with preeclampsia-associated adverse outcomes. Adverse outcomes, such as eclampsia, HELLP-syndrome but also FGR, placental abruption and intrauterine fetal deaths, are further consequences of the disease process originating in the placenta. The outcome of interest of all predictive and preventive strategies must focus on detecting and preventing these adverse events (10).
3. With the angiogenic biomarkers, substantial progress has been made to achieve a better prediction of preeclampsia-associated adverse outcomes. They are deeply rooted in the pathophysiology of the disease and have been introduced in the clinical routine in many parts of the world (25, 26). (Fig. 1).

### Angiogenic biomarkers

**Fig. 2:**
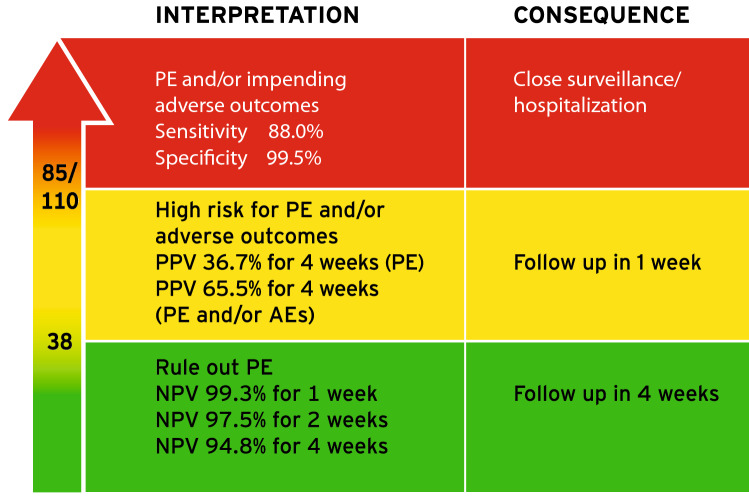
The “traffic light algorithm” of the sFlt-1/PlGF-ratio to predict preeclampsia and its adverse outcomes. Central to clinical recommendations following an sFlt-1/PlGF ratio test result and the established cut-offs of 38 and 85/110: If the sFlt-1/PlGF quotient is < 38 in a pregnant woman with suspected preeclampsia, there is a high degree of certainty that no preeclampsia or associated complications will occur within up to four weeks. For values between 38 and 85 (or 110 after 34 weeks' gestation), there is an increased risk of PE and associated complications within the next four weeks, and follow-up after one week is recommended. If the value is > 85 or 110, there is either already a manifest preeclampsia or complications are to be expected in the near future. In this case, immediate presentation to a hospital should be recommended, ideally in a perinatal center, and depending on ultrasound, CTG and laboratory findings, hospitalization may be indicated. *sFlt-1* soluble FMS-like tyrosine kinase-1, *PlGF* placental growth factor, *CTG* cardio-tocography, *PE* preeclampsia, *PPV* positive predictive value, *NPV* negative predictive value

The group of Ananth Karumanchi were the first to show that women with preeclampsia have an increased placental expression of soluble FMS-like tyrosinekinase-1 (sFlt-1) and a decreased expression of placental growth factor (PlGF) [25, 27]. Furthermore, they showed that concentrations of sFlt-1 were elevated, whereas these of PlGF were decreased in the peripheral blood of pre-eclamptic women. The degree of alteration correlated in a dose–response-like relationship to the severity of the disease: the more dysregulated placental expression and circulating concentrations in peripheral blood, the more severe the disease. These results lead to a fast development of automated tests investigating the use of the sFlt-1/PlGF ratio to improve diagnosis of the disease. In case–control studies, cut-off points for these lab results were defined that exhibited a high sensitivity and specificity to diagnose the disease [28–30]. In prospective studies, the sFlt-1/PlGF ratio showed a high predictive accuracy for preeclampsia as according to the gold standard definition [31] but also for preeclampsia-associated adverse outcomes [21, 22]. Some groups investigated the use of the single biomarker PlGF that also showed high predictive accuracy for the disease or its adverse outcomes [30, 32]. The research on how to best use sFlt-1/PlGF ratio to identify women at risk for adverse outcomes centered around the interpretation of single test results. It has been shown that repeating the test added to the predictive accuracy [33]. Time to delivery has been shown to be significantly correlated to sFlt-1/PlGF ratio levels, regardless of the presence of features of preeclampsia as defined by hypertension and proteinuria [34]. This conclusive evidence led to a widespread adoption in the clinical routine [26]. The results stemmed from well-controlled clinical studies and supported the ability of the angiogenic factors to identify women at risk. Recently, our group looked at how these study results translate into routine care. We assembled real-world evidence of the performance of the sFlt-1/PlGF ratio and were able to demonstrate that the cut-offs can be used as a reliable basis in routine work. When looking at the “traffic light scheme” (Fig. 2), the green group (sFlt-1/PlGF-ratio < 38), the yellow group (sFlt-1/PlGF-ratio ≥ 38 and < 85) and the red group (sFlt-1/PlGF-ratio ≥ 85) performed as expected with respect to outcome prediction and remaining pregnancy duration. The positive and negative predictive values of the sFlt-1/PlGF ratio at the published cut-offs were comparable in the real-world cohort and the published studies [10]. However, when applying multi-marker modeling and incorporating other clinical data together with sFlt-1 and PlGF in a regression model, we were able to enhance predictive accuracy significantly over the cut-offs as stand-alone parameters.

From a health-economic perspective, incorporation of angiogenic factor testing has the potential to save substantial costs for the payers, primarily by reducing unnecessary hospitalizations. Similar trends have been shown for many health-care systems, primarily in high resource settings [35–37]. Other works on health-economic modeling have shown that in both approaches, lowering the number of women falsely labeled as having the disease and discovering additional women that would have been overlooked otherwise has the potential to reduce costs [9, 38].

### Screening for preeclampsia

In screening for preeclampsia, advanced statistical models that provide decision support are already widely used. Especially the competing risks model of the Fetal Medicine Foundation (FMF) has improved accuracy of first-trimester screening substantially [39–43]. Their approach for early screening for preeclampsia is based on a survival time model for the time of delivery for preeclampsia. Bayes’ theorem was used to combine the prior information from maternal characteristics with biomarker multiple of the median (MoM) values. With this algorithm, screening at 11–13 weeks identifies 90% of women that will develop preeclampsia before 34 weeks and 75% of women that will develop preeclampsia before 37 weeks at a screen positive rate of 10%. The risk calculator can be accessed free of charge at the homepage of the FMF (https://fetalmedicine.org/research/assess/preeclampsia/first-trimester).

Importantly, the complex statistical modeling is superior to the risk factor-based approach [44] that is still the most frequently used modus for preeclampsia screening worldwide. Early and precise screening is of utmost importance as the prophylaxis of preeclampsia with aspirin is only effective when started before 16 weeks of gestation [45]. The ASPRE trial has shown that when screening for preeclampsia in 12–14 weeks is performed according to the FMF algorithm and aspirin is taken when risk is high, the incidence of preeclampsia was 62% lower in the intervention as compared to the placebo group [46]. The FMF competing risks model-based screening for preeclampsia in later stages of pregnancy has also shown promising results but has not yet reached widespread adoption [47–49].

### The recent change in the definition of preeclampsia

Many international and national guidelines have revised the definition of preeclampsia recently. The American College of Obstetricians and Gynecologists (ACOG) was the first to relativize the role of proteinuria and state that in the absence of proteinuria, preeclampsia can be diagnosed if in addition to hypertension other end-organ damage, such as thrombocytopenia, renal insufficiency, impaired liver function, pulmonary edema or cerebral or visual symptoms, are present [50]. In the 2013 revision of the ACOG definition, the fetal compartment has not been recognized. There is no mention of fetal consequences of poor placentation such as fetal growth restriction (FGR). This has been introduced in the revision of the definition of preeclampsia by the International Society for the Study of Hypertension in Pregnancy (ISSHP) of 2018. There, preeclampsia is defined as the new onset of hypertension in combination with end-organ symptoms, such as liver dysfunction, hemolysis, thrombocytopenia, or fetal growth restriction [51].

The mounting evidence about the use of angiogenic biomarkers has prompted their inclusion into professional guidelines [51–53]. Recently, the ISSHP has incorporated them in the definition of preeclampsia [51]. This was supported by evidence from a large prospective cohort study at two maternity hospitals in England, with a total of 15,248 women with singleton pregnancies attending a routine hospital visit at 35 + 0–36 + 6 weeks [54]. Lai et al. evaluated five different definitions of preeclampsia, some including fetal parameters and one including angiogenic biomarkers, and compared their efficacy for predicting preeclampsia-associated adverse outcomes, defined as severe maternal hypertension, major maternal morbidity, perinatal mortality or major neonatal morbidity, neonatal unit admission ≥ 48 h, and birthweight < 10th percentile. The traditional definition included women with chronic hypertension or women with new-onset of hypertension plus a new-onset of proteinuria. They compared it, among others, to the definition of the ISSHP maternal–fetal factors plus angiogenic imbalance. They found that this definition best identified the adverse outcomes; severe hypertension (40.6% [traditional] vs 66.9% [ISSHP, *P* < 0.0001], composite maternal severe adverse event (72.2% [traditional] vs 100% for all others; *P* = 0.046), composite of perinatal mortality and morbidity (46.9% [traditional] vs 71.1% [ISSHP, *P* = 0.002], neonatal unit admission for ≥ 48 h (51.4% [traditional] vs 73.4% [ISSHP, *P* = 0.001] and birthweight < 10th percentile (40.5% [traditional] vs 78.7% [ISSHP, *P* < 0.0001]. This was mainly due to addition of laboratory results but particularly because of the addition of uteroplacental dysfunction based on objective assessment of fetal growth restriction and angiogenic markers [54].

## How should we monitor high-risk women?

To prevent disease progression and adverse outcomes, pregnant women suspected of having a hypertensive pregnancy disorder should be further evaluated and potentially monitored [13, 55]. The main goals of the initial evaluation are:

- Confirmation or exclusion of elevated blood pressure.
- Assessment of the severity of hypertension as this affects management and outcome.
- Exclusion of white coat hypertension.
- To distinguish gestational hypertension from preeclampsia, as both have a different course and prognosis [12, 13, 15, 53].

White coat hypertension must be differentiated from true hypertension in pregnancy in the initial evaluation since the possible influence of white coat hypertension on a false-positive diagnosis should not be underestimated. The reported prevalence in pregnancy is up to 30% [56]. The incidence of discrepancies between blood pressures measured in the hospital and those measured at home are similar and often attributed to white coat hypertension [57, 58]. The group of pregnant women suspected of having white coat hypertension is not to be underestimated and the distinction from normotensive pregnant women is of great relevance because it may influence further treatment and monitoring during pregnancy. Pregnant women with white coat hypertension are at an increased risk of worse perinatal and maternal outcomes compared to normotensive pregnant women. Once the diagnosis is confirmed, these pregnant women need monitoring for developing preeclampsia [59].

### Maternal parameters

If women present with suspected disease, maternal symptoms must be ascertained. Specific examination must enquire the presence of upper (right) quadrant abdominal pain, headache, visual disturbances, hyper-reflexia, disturbances of consciousness, dyspnea, and bleeding tendency. The presence or absence of proteinuria is an important clinical criterion to differentiate between gestational hypertension and preeclampsia. To quantify proteinuria, first a urine dipstick can be performed as a semi-quantitative test procedure. The procedure is quick, test strips are ubiquitously available, and the result is easy to interpret. Results of negative to trace or + 1 should not be used to definitively exclude significant proteinuria since false-positive and false-negative results are possible. For a more accurate determination, either the protein–creatinine ratio from a random urine sample (> 30 mg/mmol) or the determination of the total protein amount in 24-h urine collection (> 300 mg/day) can be performed. Initial and subsequent blood tests can help indicate end-organ damage and detect HELLP-Syndrome, as a possible severe complication of preeclampsia. Table 2 shows frequently altered blood results associated with preeclampsia. These would not be expected with gestational hypertension.

**Table 2:** Laboratory parameters and changes typical of preeclampsia, adapted according to [52]

Blood count	Hemoglobin > 13 g/dl (= > 8.0 mmol/l) Hematocrit > 38% Thrombocytes < 100,000/µl
Renal values	Creatinine ≥ 0.9 mg/dl (= 79.56 μmol) Uric acid > 5.9 mg/dl (= 350 μmol/l)
Liver values	GPT (ALT) Increase ≥ 2 times the reference range GOT (AST) Increase ≥ 2 times the reference range
Coagulation	Rapid D-dimer rise as a sign of DIC
Biochemical marker	sFlt-1/PlGF ratio to exclude/confirm the diagnosis

### Fetal parameters

Identification of risk for preeclampsia-related adverse outcomes includes assessment of fetal parameters. Fetal biometry with calculation of an estimated fetal weight should be performed. In addition, in case of FGR the amniotic fluid volume and feto-placental unit, by measuring umbilical artery and uterine artery Doppler, should be assessed.

### New advances in remote monitoring

If gestational hypertension or mild preeclampsia is diagnosed, immediate action such as delivery of the fetus is not necessary in most cases. However, close monitoring is recommended to recognize a progression of the disease at an early stage and to be able to intervene accordingly. For this reason, national and international guidelines recommend that high-risk pregnant women must be monitored closely. Remote, home blood pressure monitoring is not yet routinely recommended, as the possible advantages and disadvantages have not yet been sufficiently proven by high-quality studies [60].

Many of the above-mentioned maternal parameters can now be collected easily, quickly, objectively, and reproducibly by pregnant women in their own homes, thus enabling remote monitoring. Maternal blood pressure, clinical symptoms, and protein excretion in the urine (using urine dipstick) are particularly suitable for monitoring, as these can be collected and documented by pregnant women themselves. Ongoing studies are also investigating the usefulness of remote CTG monitoring for assessing the fetal condition at home [61] or the combination of wireless CTG and automated blood pressure devices connected to a tele-monitoring platform, as in the case of the HOTEL trial (Hospital care versus Tele-monitoring in high-risk pregnancy) [62]. In their multicentre non-inferiority randomized controlled trial, van den Heuvel et al. aim at comparing the effects on patient safety, satisfaction, and cost-effectiveness of hospital care versus tele-monitoring as an obstetric care strategy in high-risk pregnancies. In their study, tele-monitoring includes wireless CTG, blood pressure monitors, daily telephone calls with a medical expert, and weekly in-hospital exams, making it very extensive and elaborately planned. The results regarding the primary outcome, a composite of adverse perinatal outcomes, are still awaited. In their case–control study “SAFE@HOME,” van den Heuvel et al. have already shown that pregnant women at increased risk for preeclampsia, as well as the healthcare system, can benefit from implementing a digital health platform into their care. Compared to a retrospective control group (*n* = 133), the number of antenatal visits (mean 13.7 vs 16.0, *p* < 0.001) and ultrasounds (6.3 vs 7.4, *p* = 0.005) was significantly lower in the SAFE@HOME group (*n* = 103). Likewise, regarding the rate of hospital admissions due to hypertension or suspected preeclampsia (2.9% versus 13.5%, *p* = 0.004). In addition, the authors observed a high level of user satisfaction with tele-monitoring and observed no differences for maternal and perinatal outcomes [63].

In recent years, an increasing number of studies have investigated the potential benefits of remote monitoring methods in pregnancy. Home blood pressure measurements of pregnant women have often been the main subject of such studies, as these seem to have great potential on monitoring and assessing the course of pregnancy, to be able to react earlier to changes and thus reduce maternal and fetal complications [60, 64–66].

The potential benefits of home blood pressure monitoring in a non-pregnant cohort are manifold and include ease of implementation and availability for the patients, reduced costs for payers through fewer visits to the doctor and less hospital stays, increased compliance and improved association with complications [67]. An increasing number of studies are investigating the possible positive effects of home blood pressure monitoring during pregnancy and in a high-risk pregnant cohort. For example, Zizzo et al. retrospectively studied the effects of home management by remote self-monitoring in 400 intermediate- and high-risk pregnancies. These were pregnancies complicated by preterm premature rupture of membranes (PPROM), FGR, preeclampsia, gestational diabetes mellitus, high-risk of preeclampsia, or a history of previous fetal or neonatal loss. The primary outcome was perinatal death, which occurred in 9 cases. None of the fetal or neonatal deaths were attributable to the home management setting but secondary to malformations, severe fetal growth restriction, extreme prematurity or PPROM. The authors conclude that remote self-monitoring in intermediate- and high-risk pregnancies seems to be a safe alternative to inpatient or frequent outpatient care [68]. Regarding home blood pressure monitoring, Perry et al. have shown that it can be effective and does not increase the risk of adverse outcomes [64]. Assessing the feasibility of a blood pressure self-monitoring intervention for managing pregnancy hypertension was the goal of the OPTIMUM-BP trial by Pealing et al. in 2019 [69].

In this randomized controlled trial, a self-monitoring of blood pressure intervention was compared to the usual care for the management of pregnancy hypertension. They found that participants persisted with the intervention for 80% or more of their time from enrollment until delivery and thus concluded that a large RCT would be feasible [69]. When analyzing the women’s and clinician’s experiences, Pealing et al. were able to show that self-monitoring of blood pressure was acceptable both to pregnant women with hypertension and their clinicians, although blood pressure variability caused uncertainty and needs to be better understood in the context of home-monitoring [70]. With regard to the question of whether self-measured blood pressure values are similarly accurate to those measured in the hospital, the study group was able to show that in the group of women with gestational hypertension, there were only small differences in systolic (mean difference 3.76 mmHg) and diastolic blood pressure (mean difference 3.27 mmHg) measurements in the hospital compared to self-monitoring [71].

This is supported by further evidence suggesting that home blood pressure readings are accurate, if validated devices are being used [72], and help identifying white coat hypertension [65]. The systematic review and meta-analysis on home blood pressure monitoring (HBPM) in the antenatal and postpartum period from Kalafat et al. aimed at investigating the safety and efficacy of home blood pressure monitoring during pregnancy. The authors included nine studies in their meta-analysis and were able to demonstrate that the use of home blood pressure monitoring in the antenatal period reduced the risk of:

- Induction of labor (OR: 0.55, 95% CI 0.36–0.82, 444 women, *I*^2^ = 0%).
- Prenatal hospital admission (OR: 0.31, 95% CI 0.19–0.49, 416 women, *I*^2^ = 0%).
- Diagnosis of preeclampsia (OR: 0.50, 95% CI 0.31–0.81, 725 women, *I*^2^ = 37%).

In addition, the number of antenatal visits was significantly less in the HBPM group (standard mean difference: − 0.49, 95% CI − 0.82 to − 0.16, 738 women, *I*^2^ = 75%). When comparing the HBPM group to conventional care regarding composite maternal, fetal or neonatal outcomes, there were no significant differences. Based on their findings, the authors conclude that HBPM is a safe and effective method for monitoring during the antenatal and postpartum period. The main limitation is the paucity of high-quality studies on this topic, which is why randomized controlled trials are needed [60].

As the advantages outweigh the potential disadvantages in most study evaluations, the ISSHP was one of the first international societies to include recommendations on the use of tele-monitoring during pregnancy in its current guideline. This includes the use of tele-monitoring for the diagnosis and differentiation of chronic hypertension, white coat hypertension and masked hypertension during pregnancy. Furthermore, the use of tele-monitoring is recommended for ongoing monitoring of any type of hypertension during pregnancy and for monitoring blood pressure in the postpartum period [51]. Monitoring in the postpartum period can help pregnant women to maintain blood pressure limits and help doctors to adapt antihypertensive therapy to the blood pressure course [73, 74].

## Machine Learning—what are these methods adding to current predictive approaches?

### What is machine-learning?

**Fig. 3:**
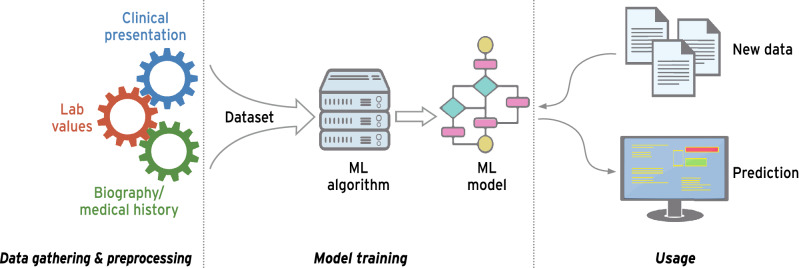
Exemplary machine-learning pipeline: The rudimentary process of creating ML model consists of three steps. Data gathering, which results in a working dataset, which in turn is used to train the ML model. The model is evaluated/used in a real-world setting to give prediction with new data as input

Machine-learning by itself signifies “a subfield of computer science that is concerned with building algorithms which, to be useful, rely on a collection of examples of some phenomenon” [75]. Generally, machine-learning can be subdivided into different operating modes, such as supervised, unsupervised, semi-supervised and reinforcement learning. The primary distinction between these forms lies in the composition of the dataset and the characteristics of the training goal. While supervised learning requires a clearly labeled dataset, meaning each example exhibits an instance of a target variable that is supposed to be predicted in future examples, unsupervised learning does not possess these clear targets but rather attempts to infer the dataset’s underlying structure. Semi-supervised learning presents a composite of both modes with just a portion of the dataset possessing a label [76]. The general approach given a dataset consists of three phases, pre-processing, model training and model usage (Fig. 3) [77].

**Fig. 4:**
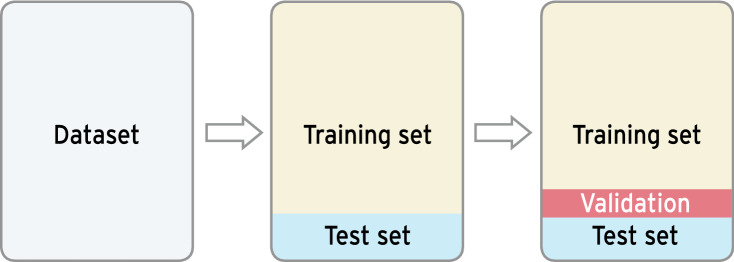
Dataset splitting visualization: Splitting of a regular dataset in training (yellow) and test (blue) data. A third dataset, the validation dataset (red) can be further split from the training dataset to determine the optimal hyper-parameters for a given model

The first phase concerns itself with establishing a consistent dataset to train and test the machine-learning algorithm with. Often, especially in medical contexts, multiple data sources, such as a hospital’s clinical information system (CIS), laboratory values, doctor’s notes and imaging data, are utilized and combined to create a single consistent dataset. Common operations are scaling and normalization [78], feature selection [79], feature encoding [80, 81], and imputation of missing values [82]. The results of these feature engineering and data cleaning operations result in a unified dataset that can be used as input to machine-learning operations. The dataset will then be divided into a training set and a test set, generally with a split in the range of 80–90% for the training dataset, depending on the size of the original collection. The training dataset is used to train the machine-learning model and establish its hyper-parameters, while the test set is used to evaluate the model’s performance on previously unseen data as an approximation for real-world performance. An additional dataset, the validation set, can be split off from the training set to evaluate the hyper-parameter tuning routines for an approximation of the test performance [76] (Fig. 4).

The training dataset is then used to train the selected algorithm and choose its hyper-parameters—meta-parameters that are not part of the algorithm’s training regimen but govern its function.

### How can we apply it to preeclampsia?

The applications in the field of preeclampsia are manifold, generally focusing on improving the detection of preeclampsia, sometimes even before the onset of symptoms. Another approach is the prediction of fetal and maternal adverse outcomes that are highly associated with the underlying risk condition established by manifest or suspected preeclampsia. Third, it is possible to assess the machine-learning models’ feature importance metrics to identify potentially highly predictive features based on a variety of data sources, patient records, doctor’s notes, laboratory values and biomarkers.

Both general approaches, prediction of preeclampsia on the one and prediction of adverse outcomes on the other side, have been explored by a multitude of different studies, especially throughout the last years.

One of the most recent works by Li et al. [83] has used the gradient-boosted trees algorithm to predict the occurrence of preeclampsia at a variety of points throughout a patient’s journey in the hospital. The established patient journeys ranging from 8 months prior to the delivery to 10 weeks, which resulted in 108,557 full pregnancy journeys, which portray 80,021 patients in total. In their work, they achieved a mean area under the curve (AUC) of 0.82 for intrapartum und 0.89 for postpartum preeclampsia. Furthermore, they performed extensive analysis regarding the importance of their collected features in the actual predictions of the algorithms.

Sandstroem et al. [84] also used electronic health records in 2019 to assess the predictive power of the random forest algorithm in comparison to two “conventional” approaches—a model following selected variables from literature (family history of preeclampsia, country of birth, method of conception, gestational age at registration, maternal age, height, weight, smoking habits, pre-existing diabetes, chronic hypertension, systemic lupus erythematodes, mean arterial pressure) and a model based on backwards feature selection by multivariate logistic regression. They performed separate analyses for preeclampsia occurrence before 34 weeks of gestation, before 37 weeks of gestation and ≥ 37 weeks of gestation. Also, they performed a separate analysis after exclusion of patients, who received treatment with aspirin. In their publication, they showed that the machine-learning algorithm performed inferior compared to the other approaches with an AUC of 0.58 compared to an AUC of 0.68 for the specified-variables model and 0.66 for the backwards selection.

Sufriyana et al. [85] explored a variety of different machine-learning models in their 2020 analysis of a 23,201-patient health insurance dataset. Their target also encompassed geographical and temporal splitting since different Indonesian regions, and their seasons exert a measurable influence on the occurrence of preeclampsia. An important part of their work was the analysis of manually entered doctor’s notes, which they used via a natural language processing (NLP) system to add additional features to the dataset.

Another target—early-onset preeclampsia—was the prediction target in the 2020 publication authored by Maric et al. [86]. They used gradient-boosted decision trees, an elastic net and logistic regression to predict preeclampsia before 34 weeks of gestation. The elastic net achieved the highest performance with an area under the curve of 0.89 and a sensitivity of 0.72.

In addition to early-onset preeclampsia, late-onset preeclampsia has also been the target of many analyses. An example is the paper published in 2019 by Jhee et al. [87]. They used a dataset of 11,006 patients and compared a multitude of different algorithms (decision trees, naive Bayes, support vector machine, random forest, stochastic gradient boosting applied on decision trees and logistic regression) of which the stochastic gradient-boosted model achieved the highest performance as measured by the ROC AUC with a value of 0.924.

Another example worth mentioning is a publication by Marin et al. [88]. Their project was based on a sensor bracelet which acted as a home monitoring device for pregnant women. Even though their dataset only consisted of 105 users, they were able to achieve an accuracy of 80% in their prediction of preeclampsia in future. This work underlines the potential of home-monitoring integration and serves as an important proof of principle.

Other works focused on the prediction of adverse outcomes in pregnancy. A recent example has been published by our working group which analyzed a dataset of 1647 women at high-risk for preeclampsia and compared two machine-learning algorithms (Random Forest, gradient-boosted trees) to cut-off-based approaches mimicking clinical decision-making [8]. The target variable was occurrence of an adverse fetal or maternal outcome at any point in future after a visit to our clinic. We were able to show that the machine-learning prediction proved superior to any cut-off-based approach on vital parameters or biomarkers such as the sFlt-1/PlGF ratio (GBtree AUC 0.81, Random Forest AUC 0.85).

A more extensive work was published in 2020 by Lipschuetz et al. [89] encompassing 9,888 patients predicted successful vaginal delivery after a previous cesarean delivery. They also showed high predictive performance ranging from an AUC of 0.76 (Random Forest) to an AUC of 0.79 (Gradient boosted trees).

Another highly interesting work, which also saw publication in 2020, was conducted by Hamilton et al. [90]. They tested a variety of different models to better determine features which are predictive for a severe neonatal morbidity. They evaluated a dataset of 1039 patients and used classification and regression trees (CART), random forest, a simple neural network, a support vector machine algorithm and generalized additive models. In their application, all models saw in fact similar performance with an AUC of about 0.85 and a sensitivity of 0.8.

The studies presented here have, to our knowledge, not been independently verified. Machine-learning performance can substantially vary when algorithms which were trained on one dataset are presented with new, independently collected data. Even though many techniques exist to reduce this so-called generalization error, only testing on new datasets can accurately assess model performance.

## Conclusion and outlook

There is an unmet medical need to improve prediction of preeclampsia-related adverse outcomes. With the advent of the angiogenic biomarkers, substantial progress has been made to better identify women at risk for adverse outcomes on the one hand, and rule out the disease despite clinical suspicion on the other hand. Professional societies have incorporated the new knowledge in revised definitions of the disease with the potential to improve care and reduce maternal and fetal morbidity and mortality. Yet, two new lines of research and clinical application show promising results to further improve care. First, remote monitoring has the potential to further reduce adverse outcomes and improve quality of life for high-risk pregnant women. Maternal blood pressure, clinical symptoms, and protein excretion in the urine, as well as fetal data, can now be collected inexpensively and reproducibly by pregnant women in their home environment and made available to the attending physicians. This has the potential to reduce unnecessary hospital visits and empower pregnant women. This is of particular importance considering the ongoing COVID pandemic. With the inclusion of signals from remote monitoring devices into predictive algorithms, home monitoring may be a fully valid alternative to a hospital visit, with respective studies and clinical trials pending. This is a main goal of future research.

We have presented examples that show that machine-learning techniques have great potential to improve the diagnostic process and treatment of preeclampsia and possibly other obstetric diseases. Though these tools show their own inherent limitations, they are superior to “traditional” statistical approaches in terms of their ability to infer complex, highly non-linear relations between statistical inputs. Due to their nature as computer-bound tools, they also are easily integrable into the existing data infrastructure present in most hospitals and highly automatable, including regular re-training and evaluation. Although prospective trials need to show their clinical efficacy in most cases, the potential for a paradigm change in medicine is enormous.

**Fig. 5:**
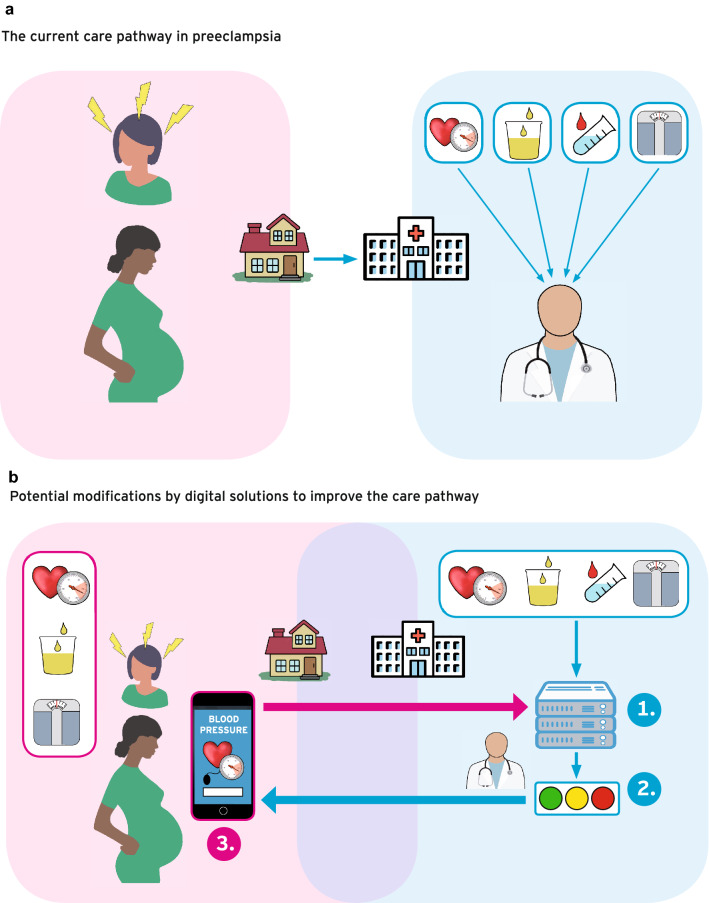
**a** The current care pathway in preeclampsia: If a pregnant woman develops signs and symptoms of preeclampsia, she will present to the hospital either independently or on the recommendation of her gynecologist or midwife. In the hospital, disease-specific symptoms are recorded, and further examinations are performed by the physician. These include measuring weight, blood pressure and protein excretion in the urine, as well as laboratory testing and ultrasound examinations. The data usually are in disconnected data silos. The physician evaluates and weighs all results to decide whether the patient is at increased risk for preeclampsia and associated complications and needs to be hospitalized or is not at increased risk and can be managed as an outpatient. This decision-making is prone to error, as it can often be influenced by many factors such as the time of day, the experience of the physician, and other external factors. **b** Potential modifications by digital solutions to improve the care pathway: Nowadays, disease-specific symptoms as well as maternal weight, blood pressure, and protein excretion in urine can be collected inexpensively and self-responsibly by patients in their homes. Digital applications allow these data to be stored and transmitted to a hospital. If a pregnant woman presents to the hospital with signs and symptoms of preeclampsia, all relevant data continue to be collected. Ideally, the data are in a data lake and accessible to predicitve algorithms that can pre-process, weigh and evaluate. The predictive algorithm feeds into decision support tools which assigns pregnant women to an appropriate risk class and can thus support the physician's decision-making

We believe that the future of our field lies in a 360° patient-centered care, with women able to input and access their data remotely into remote monitoring devices that feed into hospital-based servers. For the physicians, access to these servers and connected decision support tools will facilitate data flow over sector boundaries and ameliorate clinical decision-making (Fig. 5). An inclusion of all stakeholders, such as outpatient and hospital doctors, midwives and patients into the data flow is the road ahead. When adaptive machine-learning-based algorithms are employed to analyze the clinical data from various sources, an improved prediction of adverse outcomes has the potential to substantially improve pregnancy care.

## Data Availability

No data availability statement.
